# Exploratory analysis of mitochondrial transcription factor A as a putative biomarker for osteoporosis: a cross-sectional study

**DOI:** 10.3389/fendo.2026.1815222

**Published:** 2026-06-24

**Authors:** Yinyin Zhang, Guoying Wu, Jialu Hou, Qipei Liu, Zehua Guo, Yukai Zhang, Ying Li

**Affiliations:** 1 Third Clinical Medical College, Guangzhou University of Chinese Medicine, Guangzhou, China; 2 Endoscopy Center, Guangdong Provincial Hospital of Integrated Traditional Chinese and Western Medicine, Foshan, China; 3 Spine Department, The Third Affiliated Hospital of Guangzhou University of Chinese Medicine, Guangzhou, China

**Keywords:** mitochondrial proteins, osteoporosis, osteoprotegerin, oxidative stress, RANK ligand

## Abstract

**Background:**

Numerous studies have linked mitochondrial dysfunction to osteoporosis; however, the roles of mitochondrial transcription factor A (TFAM) and oxidative stress in osteoporosis are unclear. This exploratory cross-sectional study examined the relationship between TFAM, oxidative stress markers, and osteoporosis.

**Methods:**

This study included 48 participants aged 50–75, including 12 non-osteoporosis patients and 36 osteoporosis patients. Bone mineral density was assessed, and serum and bone samples were analyzed for TFAM, RANKL, OPG, BMP, SOD2, and MDA levels.

**Results:**

No significant differences in age, height, weight, or BMI were observed between groups. The osteoporosis group had higher serum MDA levels and lower SOD2 levels than the control group. Bone tissue analysis in the OP group revealed decreased TFAM and OPG expression and increased RANKL expression, with no consistent change in BMP across protein and mRNA levels. BMD was positively correlated with SOD2, TFAM, and OPG and negatively correlated with MDA and RANKL. In multiple regression, TFAM showed a significant association with BMD (β = 0.326, P = 0.005), but the model was limited by small sample size and risk of overfitting. The AUC for TFAM was 0.706 (95% CI: 0.52–0.86).

**Conclusion:**

TFAM may represent a candidate biomarker for osteoporosis, pending validation in larger, prospective cohorts. Causal and therapeutic potential should be addressed by interventional and functional studies.

## Graphical Abstract

**Figure:**
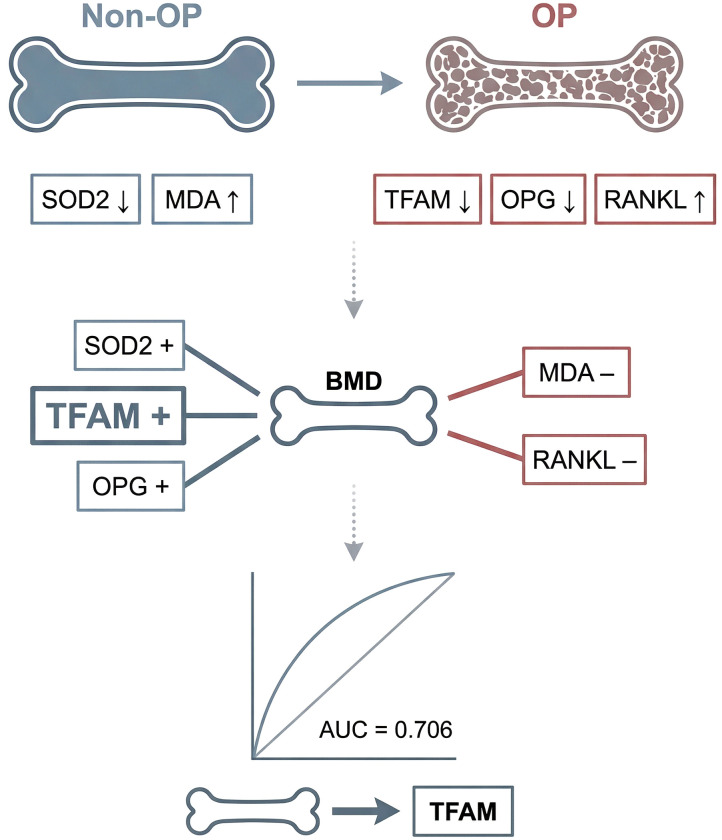

## 1 Introduction

Osteoporosis (OP) is a systemic skeletal disorder characterized by reduced bone mass and deterioration of bone microarchitecture, primarily resulting from an imbalance between bone resorption and formation. According to the World Health Organization, approximately 200 million people are affected by OP worldwide, with prevalence rates as high as 30% in women and 20% in men over the age of 50 (1). Osteoporotic fractures, the most serious complication, lead to 8.9 million cases of disability annually. Notably, hip fracture patients face a one-year mortality rate of 20%, and related medical costs constitute over 30% of global orthopedic disease expenditures (2). Although current treatments such as bisphosphonates and RANKL inhibitors can slow bone loss by inhibiting osteoclast activity, their long-term use is constrained by adverse effects, including osteonecrosis of the jaw and atypical femoral fractures (3, 4). Moreover, treatment strategies based solely on bone mineral density (BMD) T-scores fail to account for disease heterogeneity, leading to suboptimal responses in nearly 40% of patients (5, 6). These limitations highlight the need to explore novel pathogenic mechanisms of OP.

Growing evidence suggests that dysregulated bioenergetics in bone cells play a critical role in OP progression (7).Osteoblast differentiation demands substantial ATP supply, wherein mitochondria—the cellular “power plants”—directly regulate osteogenic activity through their functional integrity (8). However, current bone metabolism biomarkers such as β-CTX and P1NP mainly reflect the “outcome” of bone turnover rather than its underlying “etiology, “ particularly concerning energy metabolism dysregulation. Mitochondrial transcription factor A (TFAM), a central regulator of mitochondrial DNA (mtDNA) packaging and stability, maintains mtDNA integrity and copy number while serving as a metabolic hub (9). TFAM expression is modulated by peroxisome proliferator-activated receptor gamma coactivator 1-alpha (PGC-1α), a key coordinator of energy metabolism (10), underscoring its pivotal regulatory role. Aberrant TFAM expression has been linked to multiple age-related diseases; its deficiency promotes mtDNA damage, accelerates cellular senescence (11), and is associated with neurodegenerative, cardiovascular, and malignant disorders (12). These findings imply TFAM’s potential involvement in OP through mitochondrial regulation. In contrast to conventional biomarkers that reflect bone turnover rates, TFAM offers an upstream perspective for understanding OP pathophysiology, centered on energy metabolism and cellular senescence. Nonetheless, its dynamic expression in clinical OP contexts remains unclear. Therefore, this study aimed to investigate the relationship among TFAM, oxidative stress markers, and osteoporosis, with the goal of generating hypotheses for future mechanistic and diagnostic studies.

## 2 Materials and methods

### 2.1 Study population and sample size

This cross-sectional study consecutively enrolled 48 surgical inpatients from the Department of Orthopedics between June 2024 and December 2024. Participants were allocated based on BMD T-score (≤ -2.5 for the osteoporosis group OP, > -2.5 for the non-osteoporosis group Non-OP), resulting in an imbalanced group distribution (Non-OP, n=12; OP, n=36). This imbalance reflects the higher prevalence of osteoporosis in the inpatient surgical population; no additional matching or enrichment strategy was applied. Sample size was estimated *a priori* using G*Power (version 3.1.9.7). Based on an unpublished pilot study, an effect size (Cohen’s f) of 0.85 was assumed for the primary comparison of TFAM expression between groups. With a two-sided alpha of 0.05 and a power of 80%, the minimum required sample size was calculated as 48 participants. Of note, this effect size is large, and the calculation did not account for multivariate modeling or subgroup imbalances. Consequently, the sample is adequate for univariate comparisons but limited for multiple regression; findings should therefore be interpreted as exploratory. The proportion of patients with lumbar spinal stenosis versus degenerative spondylolisthesis was similar between the two groups (data not shown), suggesting that the observed molecular differences are unlikely to be driven by underlying spinal pathology. All participants provided written informed consent, and the study protocol was approved by the Institutional Review Board (Approval No. PJ-XS 20240521-009; May 31, 2024).

### 2.2 Inclusion and exclusion criteria

#### 2.2.1 Inclusion criteria

(1) Age ≥50 years. (2) Diagnosis of primary osteoporosis according to the World Health Organization criteria (BMD T-score ≤ -2.5 at the lumbar spine, L1-L4). (3) Scheduled for elective orthopedic surgery involving the lumbar spine (indications: lumbar spinal stenosis or degenerative spondylolisthesis, without traumatic fractures or malignancy), with cancellous bone sampling as an integral component of the surgical procedure. (4) Ability to provide informed consent and adhere to study protocols.

#### 2.2.2 Exclusion criteria

(1) Significant comorbidities, including uncontrolled cardiovascular disease (NYHA Class III/IV), diabetes mellitus with HbA1c >7.5%, severe hepatic impairment (Child-Pugh Class B or C), chronic kidney disease (stage ≥3, eGFR <60 mL/min/1.73m²), or a history of major stroke. (2) Secondary osteoporosis attributable to endocrine disorders (e.g., hyperthyroidism, Cushing’s syndrome), malabsorption syndromes, or chronic glucocorticoid use (equivalent to >5 mg/day prednisone for >3 months). (3) Active malignancy, bone metastatic disease, or acute vertebral fractures. (4) Use of bone-modifying agents (e.g., bisphosphonates, denosumab, teriparatide, or selective estrogen receptor modulators) within the preceding 6 months. (5) Cognitive impairment, psychiatric disorders, or substance abuse that would compromise the ability to provide informed consent. (6) Pregnancy or lactation.

### 2.3 Clinical data and bone mineral density assessment

Demographic and anthropometric data (age, sex, height, weight) were recorded at enrollment. Body mass index was calculated as weight (kg)/height (m)². Areal BMD (g/cm²) was measured at the lumbar spine (L1-L4) using a dual-energy X-ray absorptiometry (DEXA) scanner (QDR 4500 Discovery A, Hologic Inc., USA), with daily quality control calibration performed according to manufacturer specifications.

### 2.4 Serum biomarker analysis by ELISA

Fasting venous blood (5 mL) was collected from the antecubital vein between 06:00-08:00 after a 12-hour overnight fast. Serum was separated by centrifugation at 3, 000 × g for 15 minutes at 4 °C and stored at -80 °C until analysis. Serum levels of superoxide dismutase 2 (SOD2) and malondialdehyde (MDA) were quantified using commercial ELISA kits (MEIMIAN Co., Ltd., Jiangsu, China; Catalog# MM-0390H1 and MM-2037H1, respectively). All assays were performed in duplicate according to manufacturer protocols. Absorbance was measured at 450 nm using a Varioskan LUX multi-mode microplate reader (Thermo Fisher Scientific, USA). Standard curves were generated using four-parameter logistic regression, and sample concentrations were interpolated accordingly. Intra- and inter-assay coefficients of variation were <8% and <12%, respectively.

### 2.5 RNA extraction and quantitative PCR

Total RNA was extracted from lumbar cancellous bone specimens using RNAex Pro RNA Extraction Reagent (AGIBO, China; Cat# AG21102) according to the manufacturer’s instructions. RNA concentration and purity were assessed spectrophotometrically (AA-7000, Shimadzu, Japan); all samples exhibited A260/A280 ratios between 1.8 and 2.0. Genomic DNA was removed, and first-strand cDNA was synthesized from 1 μg of total RNA using the Evo M-MLV Reverse Transcription Pre-mix Kit (AGIBO, China; Cat# AG11728). Quantitative PCR (qPCR) was performed using the SYBR Green Pro Taq HS Pre-mixed qPCR Kit (AGIBO, China; Cat# AG11701) on a QuantStudio 5 Real-Time PCR System (Applied Biosystems). The 20-μL reaction mixture contained 10 μL of Pre-mix, 0.8 μL of primer mix (10 μM each), 2 μL of cDNA, and 7.2 μL of nuclease-free water. Thermal cycling conditions were as follows: initial denaturation at 95 °C for 30 s, followed by 40 cycles of 95 °C for 5 s and 60 °C for 30 s. Relative gene expression levels were calculated using the 2^−ΔΔCT^ method, with GAPDH serving as the endogenous control. Primer sequences are provided in **Table 1**.

**Table 1:** TFAM primer sequence.

Primer (Human)	Forward	Reverse
Actin	CATGTACGTTGCTATCCAGGC	CTCCTTAATGTCACGCACGAT
TFAM	ATGGCGTTTCTCCGAAGCAT	TCCGCCCTATAAGCATCTTGA

### 2.6 Western blot analysis

Protein was extracted from bone tissue using RIPA lysis buffer supplemented with 1× protease and phosphatase inhibitor cocktail (Biosharp; Cat# BL629B, BL615A). Protein concentration was determined using a BCA Protein Assay Kit (Biosharp; Cat# BL521A) with a microplate reader (Biotek, ELX800). Equal amounts of protein (30 μg per lane) were separated by 10% SDS-PAGE (acrylamide from Biosharp, BL513B) using a vertical electrophoresis system (TANON, VE-180B) and then transferred to a nitrocellulose membrane (Millipore, HATF00010) using a transfer tank (TANON, VE586).The membrane was blocked in TBST containing 5% skimmed milk (BioFroxx; 1172GR100) for 1 hour at room temperature and then incubated with primary antibodies against TFAM (1:1000) and GAPDH (Affinity; AF7021, 1:5000) overnight at 4 °C. After washing, the membrane was incubated with HRP-conjugated secondary antibodies (Affinity, S0001/S0002; diluted 1:5000) for 1 h at room temperature. Protein bands were visualized using an enhanced chemiluminescence substrate (Biosharp; BL520A) and imaged with a chemiluminescence instrument (TianNeng, 5200sf). Band intensities were quantified using ImageJ software (National Institutes of Health, USA). All the reagents and experimental equipment are detailed in **Supplementary File 1**.

### 2.7 Statistical analysis

Data analysis was conducted utilizing SPSS (version 26.0, IBM, US) and R (version 4.3.1) for datasets that demonstrated a normal distribution, as confirmed by the Shapiro-Wilk test. Initially, the Shapiro-Wilk test was employed to ascertain whether the data conformed to a normal distribution. For datasets adhering to normality, results were expressed as the mean ± standard deviation (x¯ ± s). Conversely, for non-normally distributed data, the median and interquartile range [M(P25~P75)] were reported. For comparative analysis between two groups, the t-test was applied when both groups exhibited normal distribution; otherwise, the Mann-Whitney U test was utilized. Correlation analysis was performed using the Pearson correlation test for normally distributed data, while the Spearman correlation test was applied for non-normally distributed data. Further analyses, including the examination of the correlation matrix, regression analysis, and ROC analysis, were executed using the R software. Statistical significance was determined using a two-tailed P-value of less than 0.05.

## 3 Results

### 3.1 Demographic characteristics

The study cohort comprised 48 participants, categorized into non-osteoporosis (Non-OP, n=12) and osteoporosis (OP, n=36) groups. The groups showed comparable baseline characteristics, with no significant differences in age, sex distribution, or anthropometric measures (all P > 0.05; age P = 0.091, sex P > 0.999, height P = 0.443, weight P = 0.637, BMI P = 0.956). Specifically, the Non-OP group (4 males, 8 females) had a mean age of 64.58 ± 10.50 years and BMI of 24.56 ± 3.38 kg/m², while the OP group (13 males, 23 females) demonstrated similar values (age 69.39 ± 7.55 years; BMI 24.64 ± 4.75 kg/m²). However, bone mineral density differed significantly between groups (Non-OP: 0.98 [0.90, 1.13] g/cm^2^; OP: 0.71 [0.57, 0.74] g/cm³; P < 0.001) (**Table 2**).

**Table 2:** Demographic and clinical characteristics at baseline.

Variable	Overall, N = 48^1^	Non-OP N = 12 (25%)^1^	OP N = 36 (75%)^1^	P-value^2^
Age(years)	68.19 (8.52)	64.58 (10.50)	69.39 (7.55)	0.091
Height(cm)	158.69 (8.47)	160.33 (10.07)	158.14 (7.95)	0.443
Weight(kg)	61.85 (10.96)	63.17 (10.69)	61.42 (11.17)	0.637
BMI(kg/m²)	24.62 (4.42)	24.56 (3.38)	24.64 (4.75)	0.956
BMD(g/cm2)	0.73 [0.65, 0.80]	0.98 [0.90, 1.13]	0.71 [0.57, 0.74]	<0.001
Sex				> 0.999
Male, No. (%)	17 (35.42%)	4 (33.33%)	13 (36.11%)	
Female, No. (%)	31 (64.58%)	8 (66.67%)	23 (63.89%)	

¹Data are presented as n (%), mean (SD), or median [IQR].

²Statistical comparisons were performed using Fisher's exact test, two-sample t-test, or Wilcoxon rank sum test, as appropriate.

### 3.2 Systemic oxidative stress in osteoporosis

**Figure 1:**
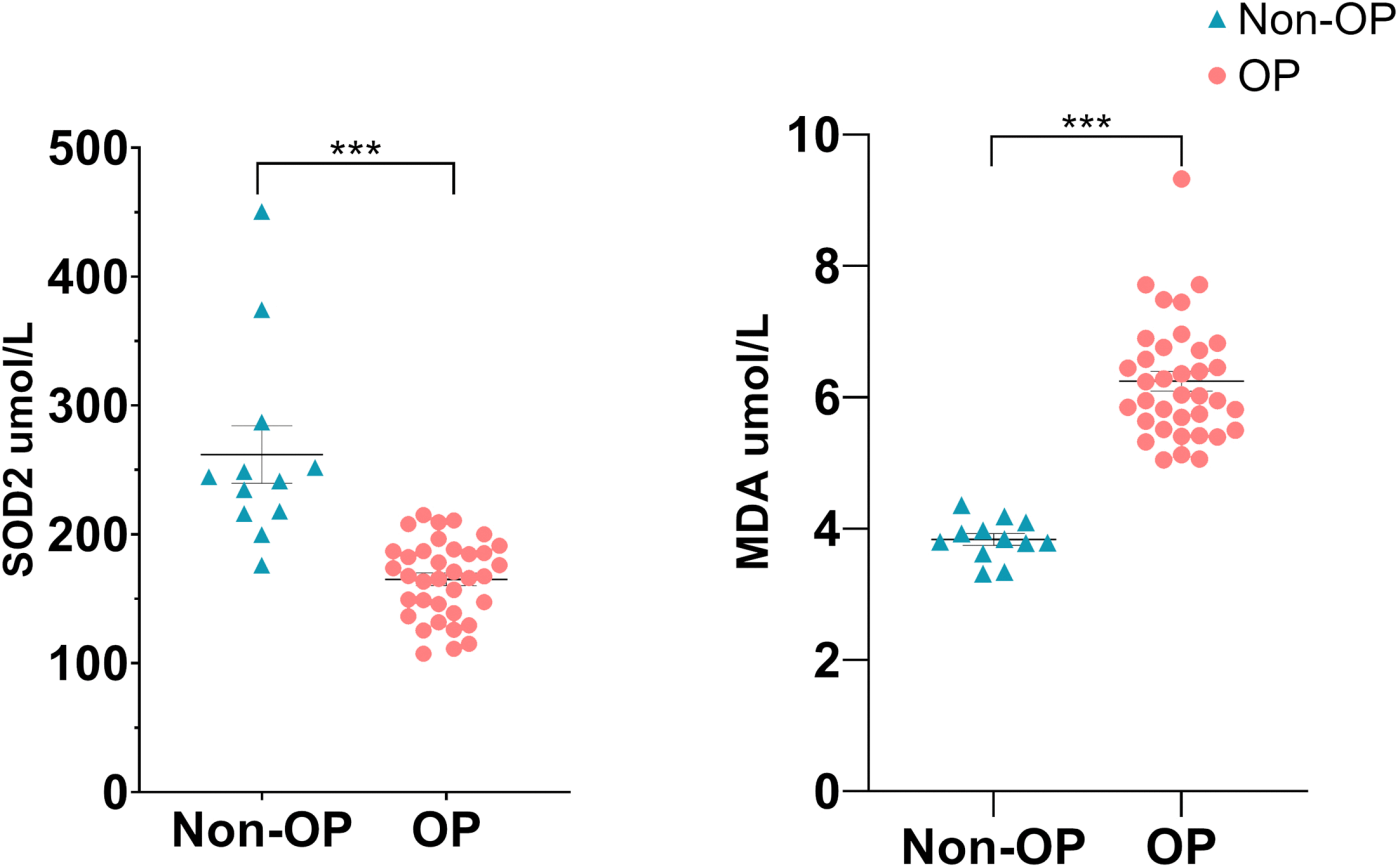
Serum levels of oxidative stress markers in OP and Non-OP groups serum concentrations of superoxide dismutase 2 (SOD2) and malondialdehyde (MDA) in the OP (n=36) and Non-OP (n=12) groups. Data are presented as mean ± SEM. Statistical significance was determined by independent Student's t-test. *P < 0.05, **P < 0.01, ***P < 0.001.

Serum levels of malondialdehyde (MDA) and superoxide dismutase 2 (SOD2) were measured by enzyme-linked immunosorbent assay (ELISA). Compared with the Non-OP group, the OP group showed significantly decreased SOD2 levels and increased MDA levels (both P < 0.001) (**Figure 1**).

### 3.3 Mitochondrial dysfunction and bone remodeling imbalance

**Figure 2:**
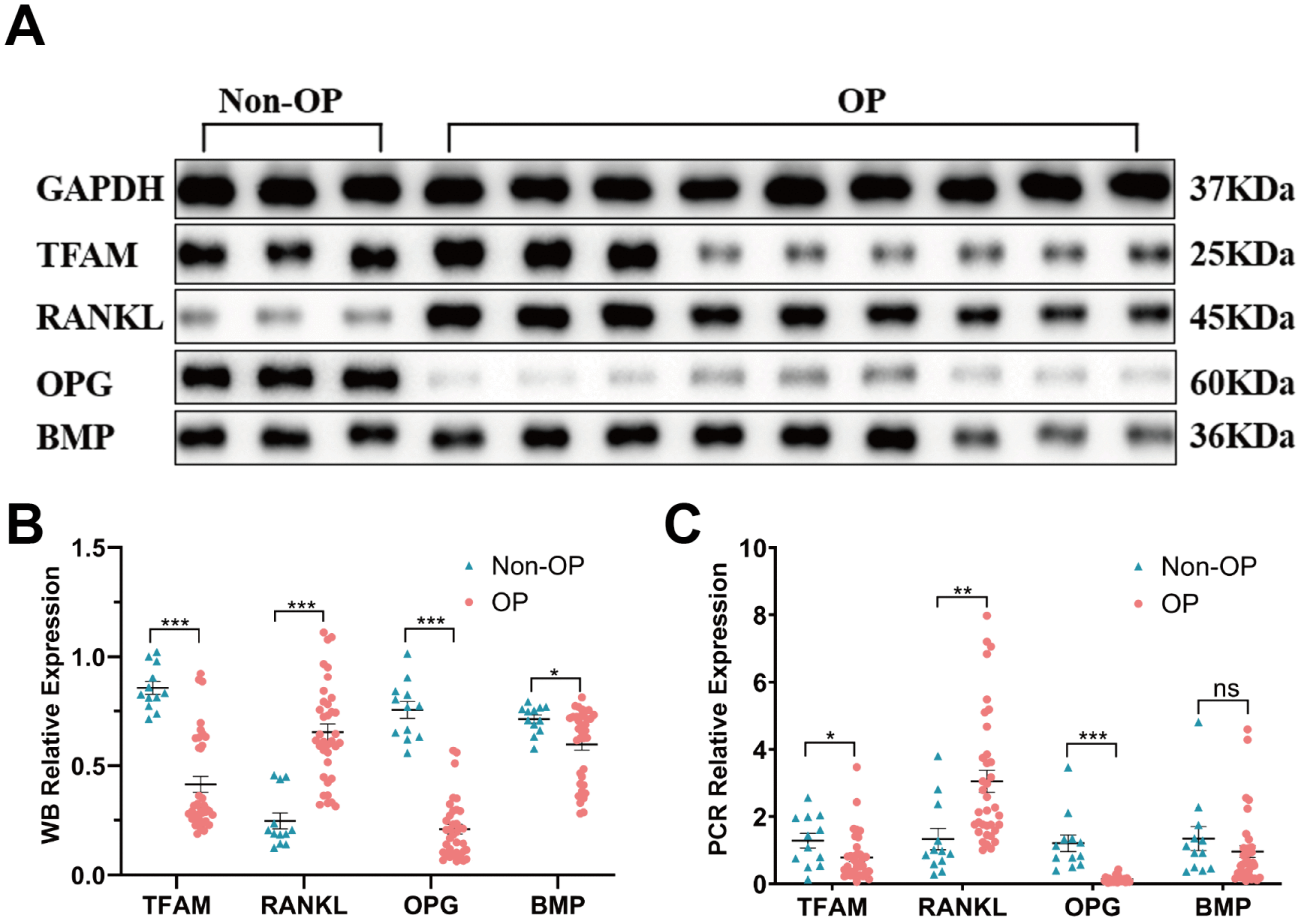
Protein and mRNA expression of bone metabolism markers in OP and non-OP groups **(a)** Representative Western blot images showing protein expression of TFAM, RANKL, OPG, and BMP in bone tissue extracts from OP and Non-OP groups. GAPDH served as a loading control. Each group includes four representative samples, with three biotechnological replicates per sample. **(b)** Relative protein expression levels of TFAM, RANKL, OPG, and BMP in OP (n=36) and Non-OP (n=12) groups. Data are presented as mean ± SEM. **(c)** Relative mRNA expression levels of TFAM, RANKL, OPG, and BMP in OP (n=36) and Non-OP (n=12) groups. Data are presented as mean ± SEM. Statistical significance was determined by independent Student's t-test. *P < 0.05, **P < 0.01, ***P < 0.001; ns, not significant.

Western blot analysis revealed differential protein expression in bone tissue between the two groups (**Figure 2a**; Original image in **Supplementary File 2**). Compared with the Non-OP group, the OP group showed significantly decreased TFAM and OPG levels, along with increased RANKL expression (all P < 0.001). To further evaluate the net osteoclastogenic potential, we calculated the RANKL/OPG ratio for each participant based on their mRNA expression levels measured by qPCR. The OP group exhibited a markedly elevated RANKL/OPG ratio (23.66 [14.24, 43.77]) compared to the Non-OP group (1.27 [0.48, 2.42]), with the difference being highly significant (Wilcoxon rank sum test P < 0.001). These protein-level findings were consistent with qPCR results. In contrast, while BMP protein expression was significantly lower in the OP group by western blot (P = 0.030), no such difference was observed at the mRNA level (P = 0.114) (**Figures 2b, c**). All detailed statistical data are provided in **Supplementary File 3**.

### 3.4 Correlation and predictive modeling of BMD determinants

**Figure 3:**
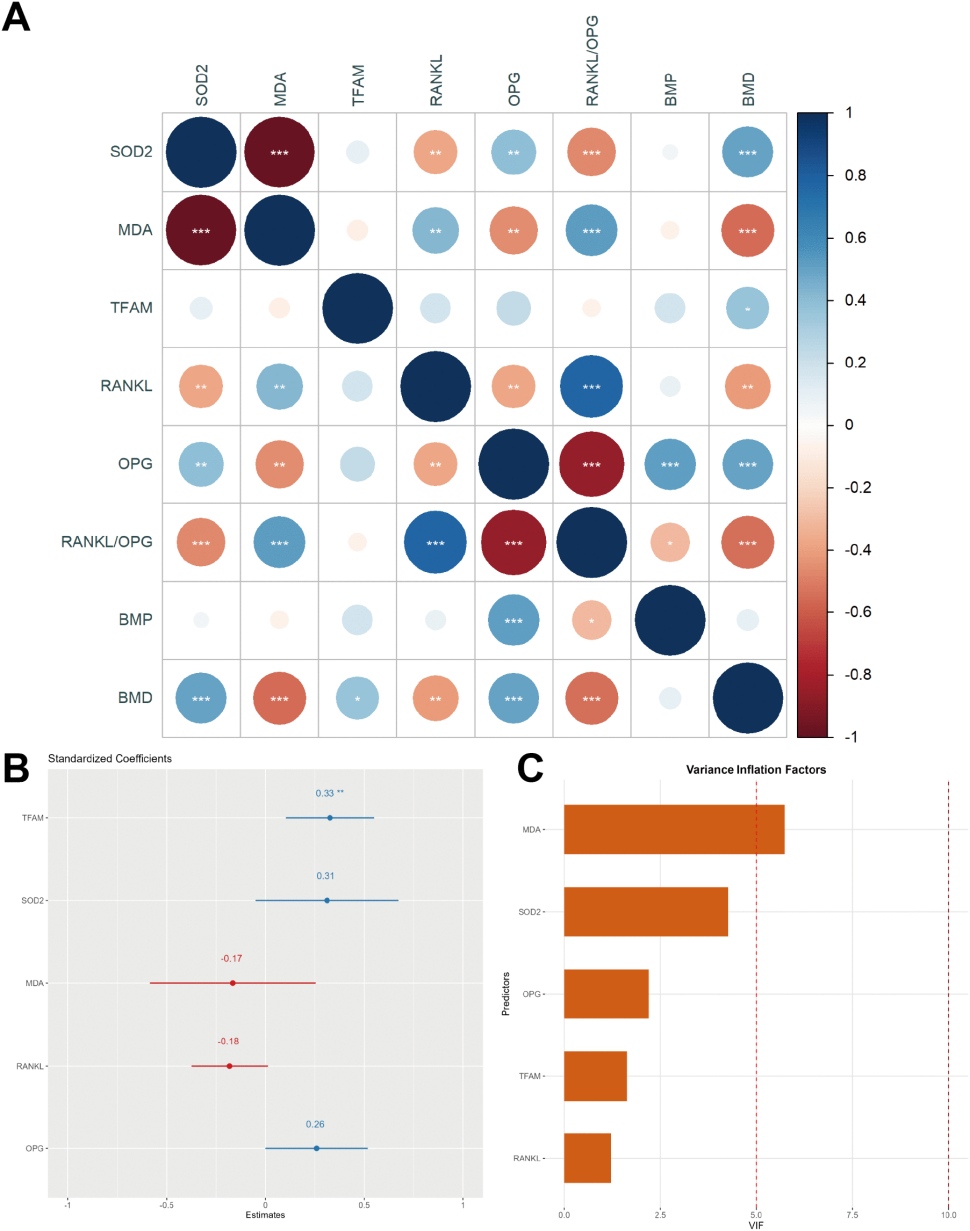
Correlation and regression analyses of bone metabolism markers **(a)** Correlation matrix heatmap depicting the relationships among biomarkers. Circle size and color intensity reflect correlation strength. Red indicates negative correlation, and blue indicates positive correlation. *P < 0.05, **P < 0.01, ***P < 0.001. **(b)** Forest plot visualizing the multiple regression model results. Blue squares represent protective factors (positive association with BMD), and red squares represent risk factors (negative association with BMD). Horizontal lines indicate 95% confidence intervals. *P < 0.05, **P < 0.01. **(c)** Variance inflation factor (VIF) analysis for assessment of multicollinearity among variables. All VIF values <10 indicate no significant collinearity.

We analyzed biomarker correlations with BMD using Spearman’s rank correlation test (**Figure 3a**; see matrix in **Supplementary File 4**). SOD2, TFAM, and OPG showed significant positive correlations with BMD (SOD2: r = 0.65, P < 0.001; TFAM: r = 0.44, P = 0.0016; OPG: r = 0.66, P < 0.001). MDA and RANKL demonstrated significant negative correlations with BMD (MDA: r = -0.63, P < 0.001; RANKL: r = -0.35, P = 0.016). The RANKL/OPG ratio exhibited a moderate negative correlation with BMD (r = -0.39, P = 0.006) and a significant positive correlation with the oxidative stress marker MDA (r = 0.37, P = 0.009). BMP was not significantly correlated with BMD (r = 0.13, P = 0.38). Complete correlation matrix results are provided in **Supplementary File 4**.

A multiple regression model incorporating the biomarkers (SOD2, MDA, TFAM, RANKL, OPG) was statistically significant (F = 18.170, P < 0.001) and explained 64.6% of the variance in BMD (adjusted R² = 0.646; **Table 3**). Among these predictors, TFAM showed a significant positive association (β = 0.326, P = 0.005). However, given the small sample size (n=48) and five predictors, this model is at risk of overfitting. The other variables—SOD2 (β = 0.312, P = 0.089), MDA (β = -0.165, P = 0.431), RANKL (β = -0.181, P = 0.066), and OPG (β = 0.259, P = 0.051)—did not reach statistical significance in the multivariate model (**Figures 3b, c**). These findings should be interpreted as exploratory.

**Table 3:** Multiple regression results.

Variable	B	β	t	P	VIF	F	Adjust R^2^
SOD2	0.001	0.312	1.740	0.089	4.258	18.170	0.646
MDA	-0.023	-0.165	-0.795	0.431	5.731		
TFAM	0.082	0.326	2.951	0.005	1.625		
RANKL	-0.018	-0.181	-1.891	0.066	1.222		
OPG	0.076	0.259	2.012	0.051	2.196		

This regression model includes five predictors with a sample size of 48, which may lead to overfitting. Results should be interpreted as exploratory.

### 3.5 Diagnostic performance of TFAM

**Figure 4:**
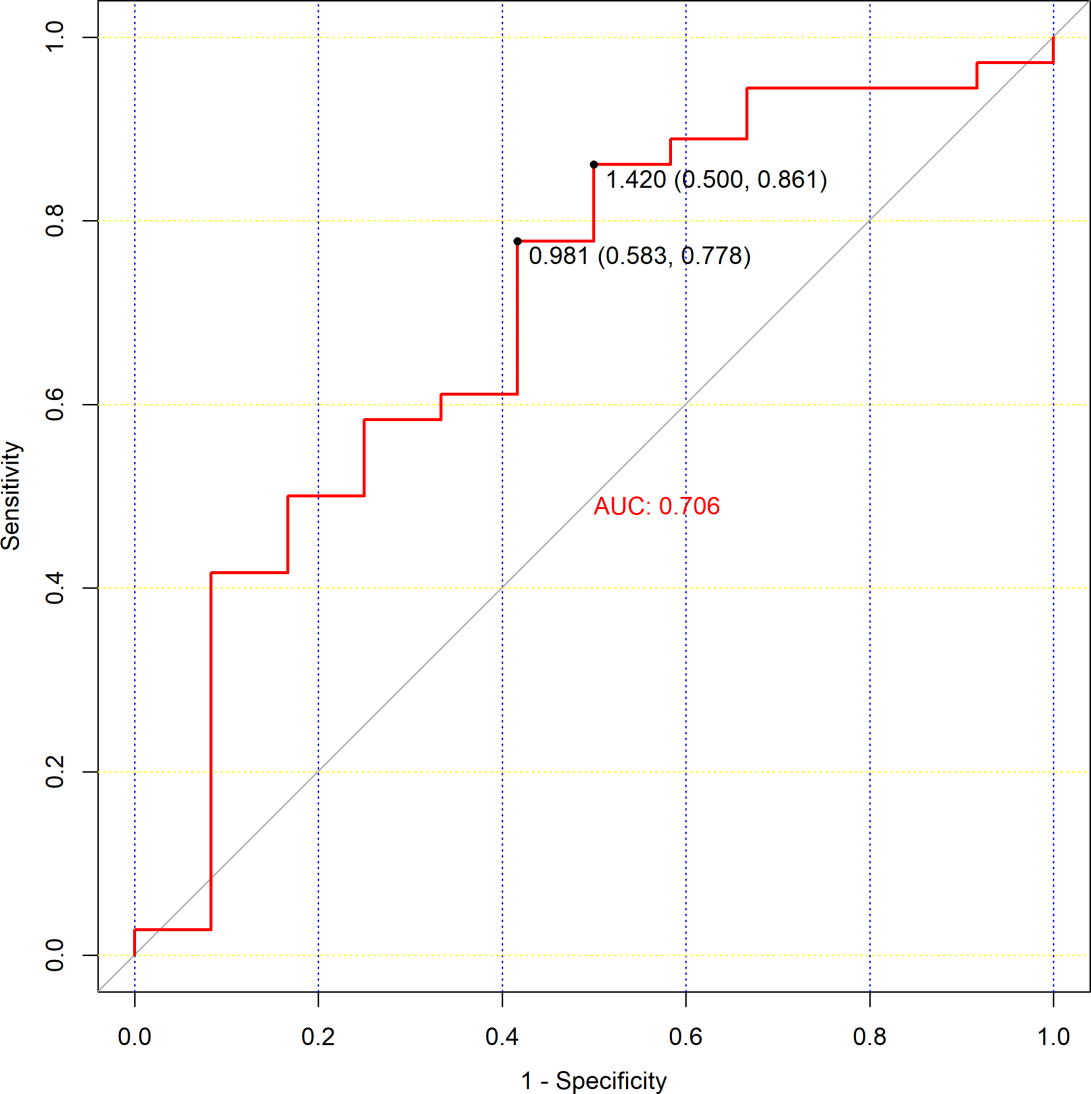
Receiver operating characteristic curve (ROC) of TFAM for osteoporosis prediction ROC curve evaluating the diagnostic performance of TFAM in distinguishing OP patients from non-OP controls. Sensitivity is plotted against 1-specificity (false positive rate). The diagonal dashed line represents the line of no discrimination (AUC = 0.5). The area under the curve (AUC) quantifies the overall predictive accuracy of TFAM.

The overall AUC was 0.706 (95% CI: 0.52–0.86), indicating moderate discriminatory power but with poor precision (lower bound of CI approaching 0.5). Bootstrap validation with 1, 000 resamples yielded similar AUC estimates (mean AUC = 0.702, 95% CI: 0.51–0.85), confirming the robustness of the point estimate but also underscoring its limited precision. Two optimal cut-off values were identified: at a relative expression of 1.420, sensitivity was 86% (95% CI: 0.86–1.00) and specificity was 50% (95% CI: 0.50–0.75); at 0.981, sensitivity was 78% (95% CI: 0.78–0.97) and specificity was 58% (95% CI: 0.58–0.83). Both cut-offs yield suboptimal specificity, which limits the clinical utility of TFAM as a standalone diagnostic test. As TFAM is expressed relatively, values near 1.0 indicate minimal intergroup differentiation; thus, the 0.981 cut-off may hold limited clinical relevance (**Figure 4**).

## 4 Discussion

To our knowledge, this is the first study to explore TFAM as a candidate biomarker for osteoporosis, demonstrating a significant positive correlation with BMD (r = 0.44, P = 0.0016). However, due to the cross-sectional design, no causal relationships can be inferred. The mechanistic scenarios discussed below are hypothetical and derived from prior literature; the present study did not perform functional assays (e.g., ROS measurement in bone tissue, TFAM knockdown/overexpression) to directly test these pathways. Therefore, the following interpretation should be viewed as exploratory and hypothesis-generating. While the diagnostic potential of TFAM is supported by an area under the curve of 0.706, its moderate discriminatory capacity—and particularly the wide confidence interval (95% CI: 0.52–0.86)—suggests it is more suitable as a candidate biomarker rather than a standalone clinical diagnostic tool.

We hypothesize that the role of TFAM in osteoporosis is fundamentally linked to its function as a master regulator of mitochondrial integrity and bioenergetics. Adequate TFAM expression is crucial for maintaining the metabolic equilibrium that governs bone remodeling, a process requiring precise functional coupling between osteoblasts and osteoclasts. Specifically, TFAM-dependent mitochondrial transcription is essential for oxidative phosphorylation, which supplies the ATP necessary for osteoblast differentiation and matrix mineralization. Concurrently, the metabolic flexibility required for osteoclast differentiation, which is heavily reliant on glycolysis, also depends on functional mitochondria safeguarded by TFAM (13). Furthermore, mitochondrial metabolic reprogramming directs the fate of bone marrow mesenchymal stem cells toward either osteogenic or adipogenic lineages, a process exemplified by the Wnt/β-catenin pathway (14).

A key pathological consequence of TFAM deficiency is the disruption of this energy homeostasis, leading to a self-perpetuating cycle of oxidative stress. As TFAM ensures the proper assembly and function of the electron transport chain, its deficiency impairs electron transfer, resulting in mitochondrial reactive oxygen species overproduction. Our data support this oxidative stress axis, as the osteoporotic group exhibited significantly higher levels of the lipid peroxidation marker malondialdehyde and lower levels of the antioxidant enzyme superoxide dismutase 2. This aligns with the known mechanisms by which ROS disrupt bone homeostasis: by inhibiting the master osteoblast differentiation factor Runx2 via JNK and FoxO1 signaling (15), and by promoting osteoclast activity. Specifically, ROS can act as a critical intracellular signaling messenger that potentiates RANKL-induced signaling pathways, such as the activation of NF-κB and c-Fos, which are essential for osteoclast differentiation and activation (15). The clinical relevance is underscored by studies showing that elevated ROS in the bone marrow microenvironment correlates with reduced osteoblast activity and increased marrow adiposity in osteoporosis patients (16). The situation is compounded by deteriorating mitochondrial quality control; defects in mitophagy can trigger osteoblast apoptosis (17), while an imbalance in mitochondrial fusion and fission, often marked by downregulation of MFN2, impairs the cellular response to mechanical loading (18). Although our study did not observe intergroup differences in bone morphogenetic protein expression—potentially due to sample size limitations—the established osteoprotective role of bone morphogenetic proteins (19) suggests that the interplay between mitochondrial function and anabolic signaling pathways warrants further investigation. Of note, although BMP protein expression was significantly lower in the OP group by western blot, no significant difference was observed at the mRNA level. This discrepancy may reflect post-transcriptional regulation or limited statistical power due to the small sample size; therefore, the finding should be interpreted with caution and requires validation in larger cohorts.

Our findings also directly address the interplay between oxidative stress and the RANKL/RANK/OPG system, a central pathway in bone resorption. The upregulated RANKL and downregulated OPG expression observed in our OP cohort, along with their correlation with MDA levels, provides clinical evidence for an association between oxidative stress and the resorption axis. This is further substantiated by the markedly elevated RANKL/OPG ratio in the OP group (P < 0.001), which integrates the net pro-resorptive shift. The moderate negative correlation between the RANKL/OPG ratio and BMD (r = -0.39, P = 0.006) underscores its potential as a composite indicator of bone resorption activity. Moreover, the positive correlation of this ratio with MDA (r = 0.37, P = 0.009) links systemic oxidative stress to enhanced osteoclastogenic potential. The elevated systemic oxidative stress (indicated by high MDA and low SOD2) in our OP cohort may serve as a driver of this imbalance. This supports the concept that ROS can potentiate RANKL signaling and, conversely, that oxidative modifications can impair OPG function (20, 21). In this context, TFAM may act as an upstream regulator; its deficiency could initiate mitochondrial ROS production that fuels this cascade. This mechanistic link, though speculative without direct functional evidence, reinforces the potential of targeting mitochondrial function as a therapeutic strategy. Furthermore, excess mitochondrial ROS can activate pro-inflammatory transcription factors like NF-κB (22), which not only directly promotes osteoclast genesis but also stimulates the production of inflammatory cytokines that further exacerbate bone resorption, creating a vicious cycle.

While this study is the first to explore TFAM as a predictive biomarker for osteoporosis, its limitations must be acknowledged. The cross-sectional design precludes conclusions about causality, and the relatively small sample size, with a predominance of female participants, introduces potential selection bias and limits the generalizability of our findings. Moreover, critical confounders including menopausal status, comorbid diabetes or chronic kidney disease, and use of medications beyond bone-modifying agents were not recorded and thus could not be controlled. Furthermore, all participants were recruited from an orthopedic surgical inpatient population undergoing spinal surgery. This convenience sampling may overrepresent severe or complicated cases and limit the generalizability of our findings to the broader community-dwelling osteoporotic population, particularly those with mild disease or managed conservatively. Second, the modest sample size-–confirmed by *post-hoc* power analysis indicating reduced sensitivity for detecting small effect sizes-–may have limited our ability to detect additional significant associations. Third, the cross-sectional nature of our data prevents establishing temporal relationships between TFAM expression and bone loss. Fourth, while we observed significant alterations in oxidative stress markers, this study did not assess systemic inflammatory markers such as TNF-α or IL-6. Given the intricate crosstalk between oxidative stress and inflammation in bone metabolism (23), future studies should incorporate a comprehensive panel of inflammatory cytokines to fully delineate the interplay between mitochondrial dysfunction, oxidative stress, and inflammation in osteoporosis. Future research should prioritize prospective cohort studies with larger, more diverse populations to validate TFAM’s predictive value. Crucially, interventional studies are needed to establish causality. *In vitro* experiments modulating TFAM expression in osteoblasts and osteoclasts could elucidate its direct effects on cell activity and differentiation. Furthermore, conditional TFAM knockout animal models would be invaluable for dissecting its specific role in bone metabolism *in vivo*, potentially revealing TFAM not only as a biomarker but also as a novel therapeutic target for mitigating age-related bone loss.

## 5 Conclusion

This cross-sectional study explored the relationship between osteoporosis and serum/bone tissue biomarkers. The principal finding demonstrated that, compared with the non-osteoporosis group, osteoporosis patients exhibited significantly altered bone tissue TFAM expression, systemic oxidative stress status, and bone turnover biomarker profiles. These findings provide novel insights into the molecular mechanisms underlying osteoporosis and identify a promising panel of biomarker combinations. TFAM may serve as a potential biomarker linking mitochondrial dysfunction to bone metabolism dysregulation. Although the cross-sectional design limits causal inference, this study establishes a foundation for future research. Subsequent investigations should prioritize external validation in independent, large-scale cohorts and undertake longitudinal studies to assess the translational potential of these biomarkers in predicting disease progression and facilitating early diagnosis.

## Data Availability

The original contributions presented in the study are included in the article/**Supplementary Material**. Further inquiries can be directed to the corresponding author.
