# Supplementary material for: Exploratory analysis of mitochondrial transcription factor A as a putative biomarker for osteoporosis: a cross-sectional study
Source: Front Endocrinol (Lausanne). 2026 Jun 24;17:1815222. doi: 10.3389/fendo.2026.1815222 (PMC13341557; doi:10.3389/fendo.2026.1815222)
Supplement: Supplementary file 1 [file DataSheet1.docx]

Supplementary Material

# Supplementary Files

**Supplementary File 1: List of Reagents and Experimental Equipment**

|  | **Name** | **Manufacturer** | **Product ID** |
| --- | --- | --- | --- |
| ELIS | SOD2 ELISA Research Kit | MEIMIAN Co., Ltd. (JiangSu, China) | MM-2037H1 |
|  | MDA ELISA Research Kit | MEIMIAN Co., Ltd. (JiangSu, China) | MM-0390H1 |
| PCR | Enzyme-free water | LABGIC Co., Ltd. (BeiJing, China) | BL510B |
|  | AG RNAex Pro RNA extracting reagent | AGIBO Co., Ltd. (HuNan, China) | AG21102 |
|  | PBS | SERVICEBIO Co., Ltd. (WuHan, China) | G4207-500ML |
|  | Analysis of pure trichloromethane | Guangzhou Chemical Reagent Factory | GD10-AR-0.5L |
|  | Analysis of pure isopropanol | Da Mao Co., Ltd. (TianJIin, China) | 2351 |
|  | Analysis of pure anhydrous ethanol | Da Mao Co., Ltd. (TianJIin, China) | 2335 |
|  | Evo M-MLV Reverse transcription pre-mix kit | AGIBO Co., Ltd. (HuNan, China) | AG11728 |
|  | SYBR Green Pro Taq HS Pre-mixed qPCR reagent kit | AGIBO Co., Ltd. (HuNan, China) | AG11701 |
|  | Steady Pure Universal type gene group DNA extraction kit box | AGIBO Co., Ltd. (HuNan, China) | AG21009 |

**Western blot analysis**

1. **experimental apparatus**

| **Name** | **Manufacturer** | **Model** |
| --- | --- | --- |
| microplate reader | biotek | ELX800 |
| Desktop high-speed refrigerated centrifuge | SCILOGEX | CF1524R |
| Shaking incubator | SCILOGEX | SLK-O3000-S |
| Vertical electrophoresis tank | TANON | VE-180B |
| Transfer electrophoresis tank | TANON | VE586 |
| Electrophoresis apparatus | LIUYI | DYY-6C |
| Chemiluminescence instrument | TIANNENG | 5200sf |

2. **Main experimental reagents**

| **Reagent** | **Manufacturer** | **Product ID** |
| --- | --- | --- |
| cooktail | Biosharp | BL629B |
| PMSF（100mM） | Biosharp | BL507A |
| phosphatase inhibitors 100× | Biosharp | BL615A |
| BCAProtein Quantitative Detection Kit | Biosharp | BL521A |
| Acrylamide/methyl vinyl 29:1, 30% solution | Biosharp | BL513B |
| Protein Ladder | Thermofisher | 26616 |
| Nitrocellulose membrane | Millipore | HATF00010 |
| Skimmed milk powder | BioFroxx | 1172GR100 |
| TWEEN 20 | BioFroxx | 1247ML100 |
| Ultra-sensitive ECL chemiluminescence substrate | Biosharp | BL520A |
| GAPDH Antibody | Affinity | AF7021 |
| Goat Anti-Rabbit IgG (H+L) HRP | Affinity | S0001 |
| Goat Anti-Mouse IgG (H+L) HRP | Affinity | S0002 |
| Anhydrous ethanol | Xilongs | 1170 |
| Tris-base | Biosharp | BS083 |

**Supplementary File 2: Original Western Blot Images**

BMP-1




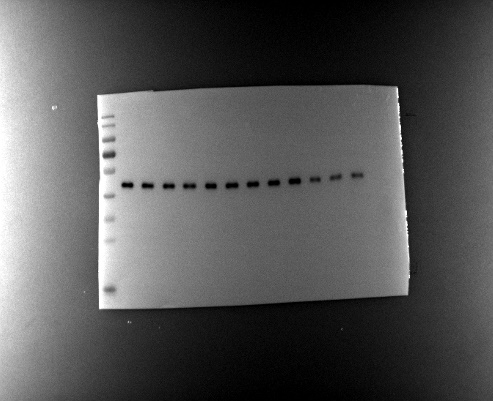

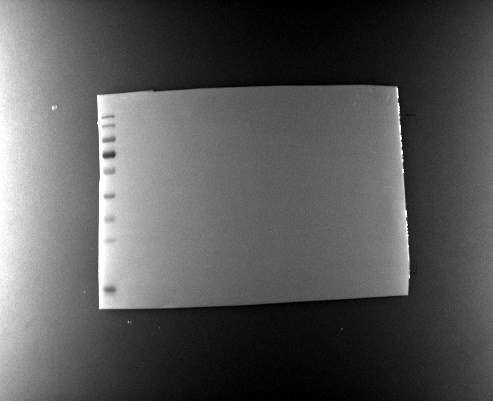


BMP-2




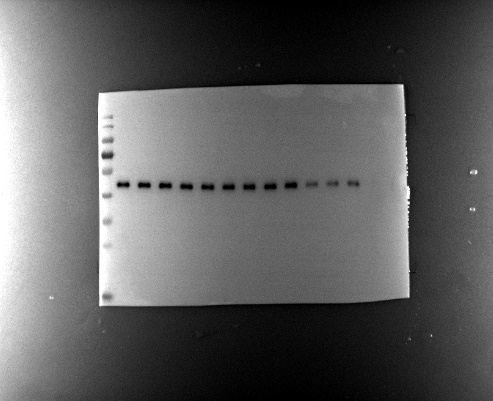

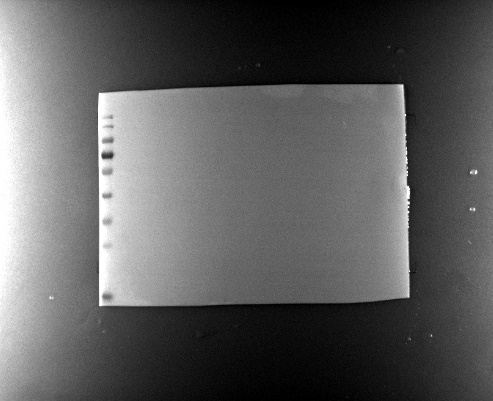


BMP-3


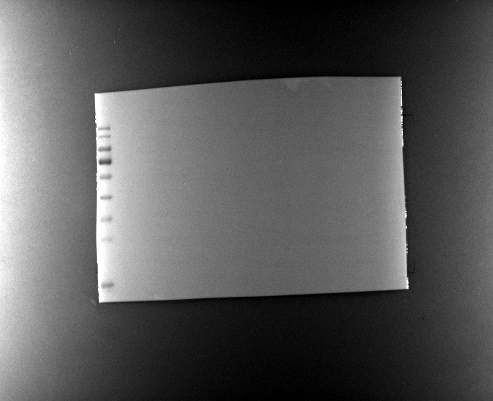

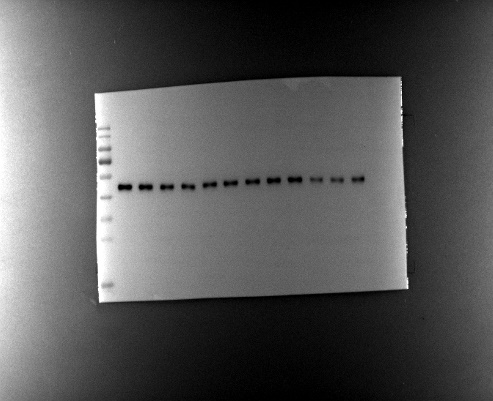




BMP-3


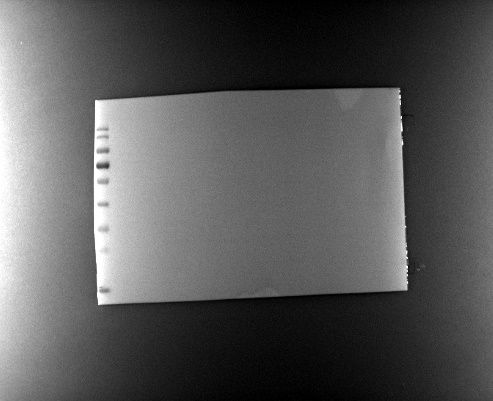

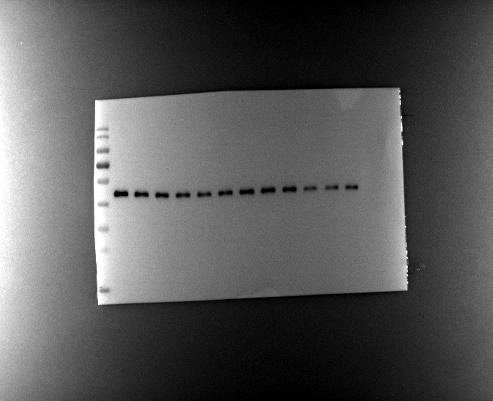




GAPDH-1


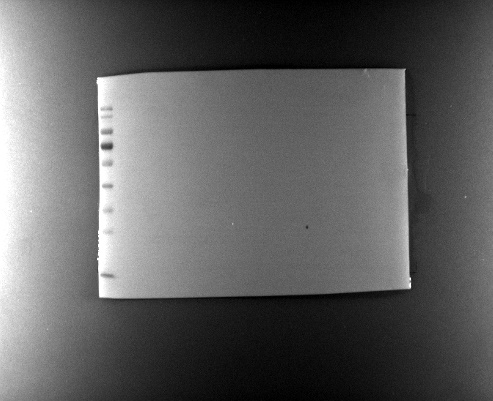

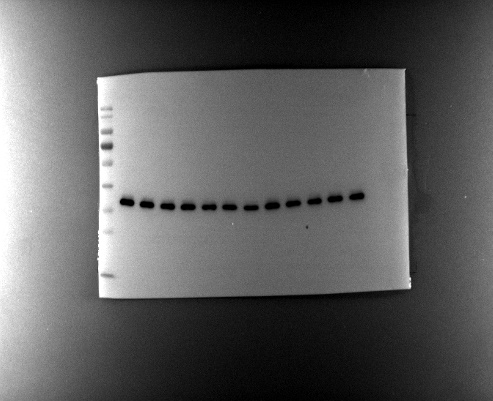




GAPDH-2


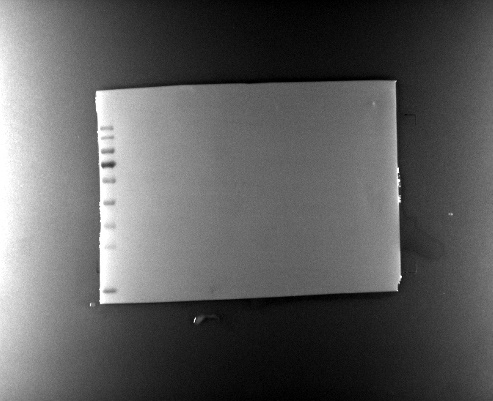

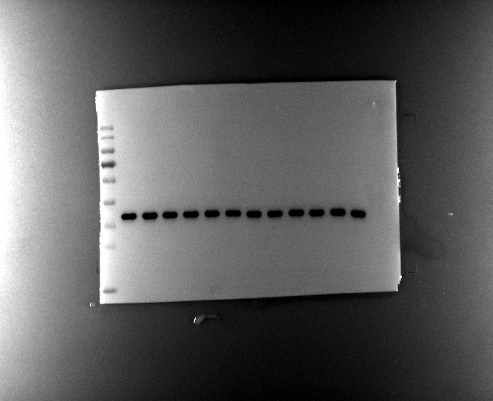




GAPDH-3


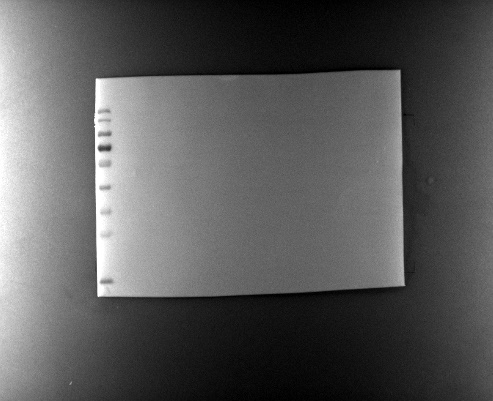

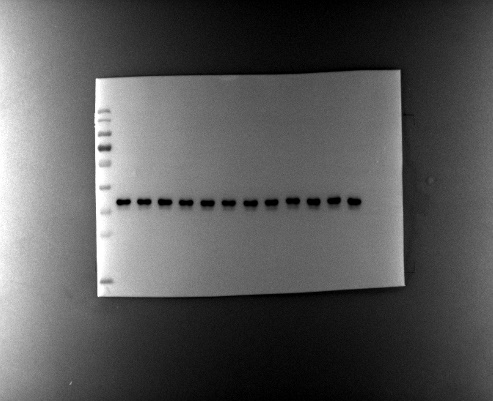




GAPDH-4


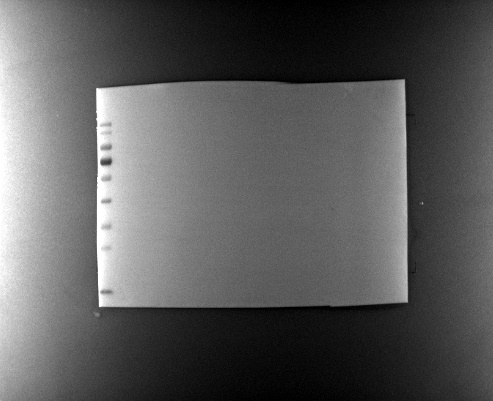

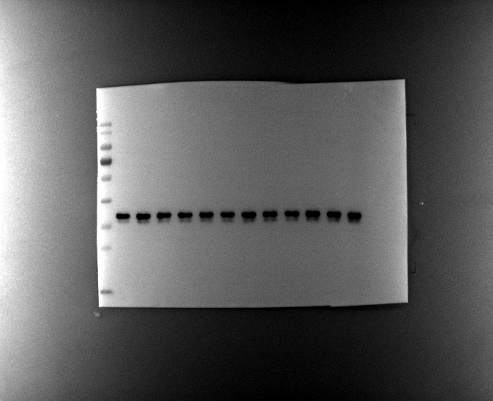




OPG-1


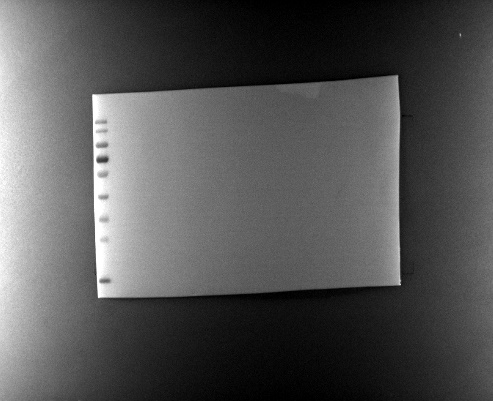

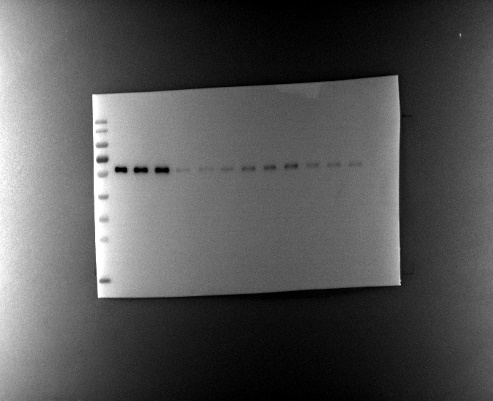




OPG-2


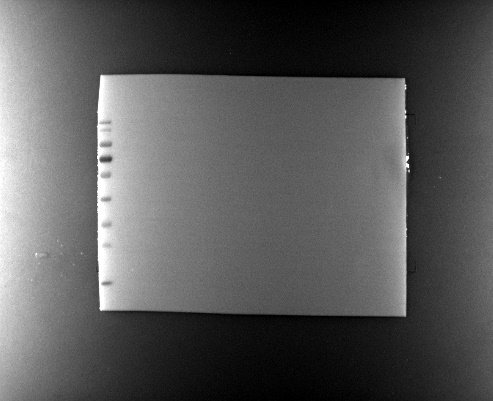

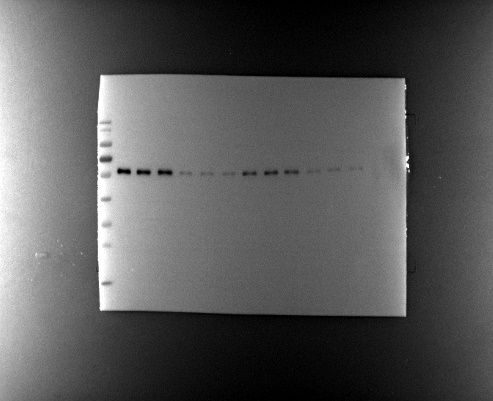




OPG-3


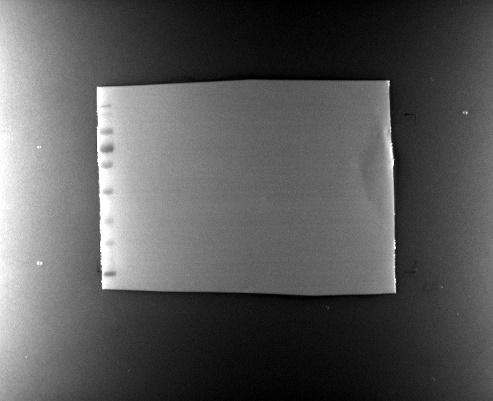

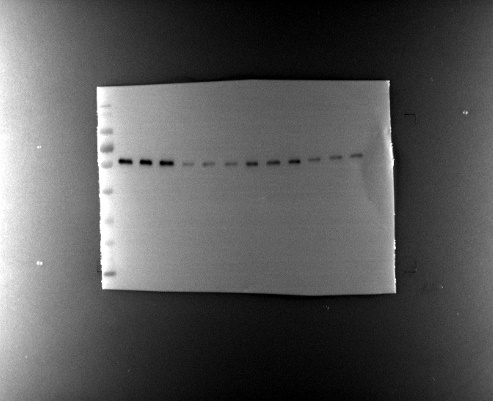




OPG-4




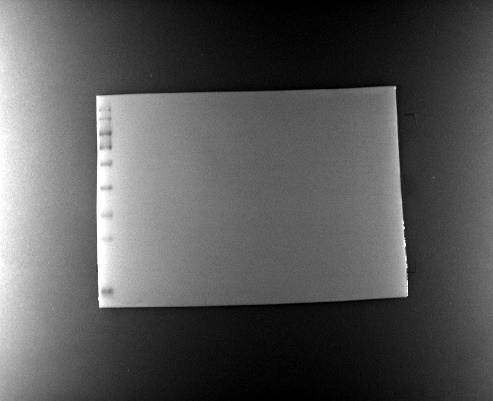

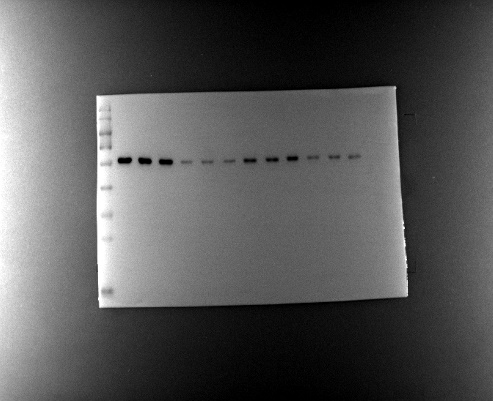


RANKL-1




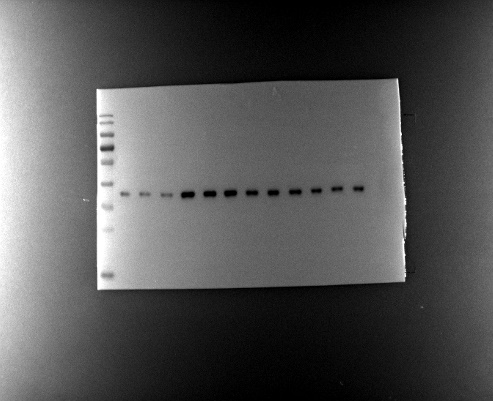

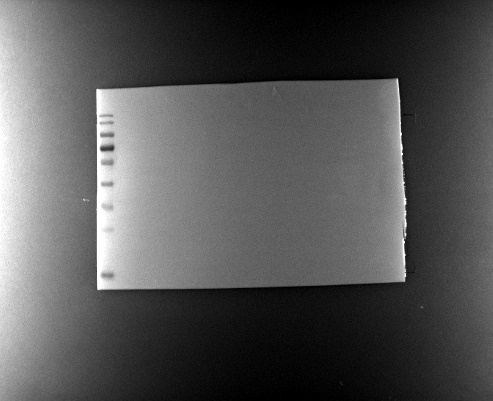


RANKL-2




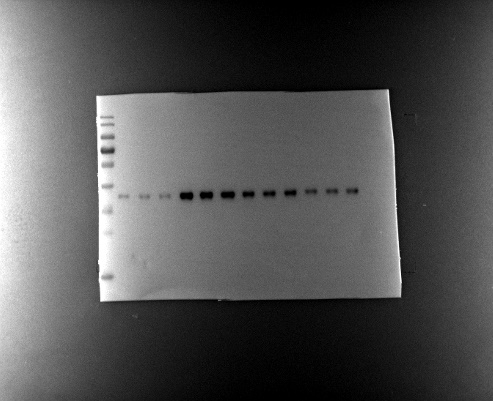

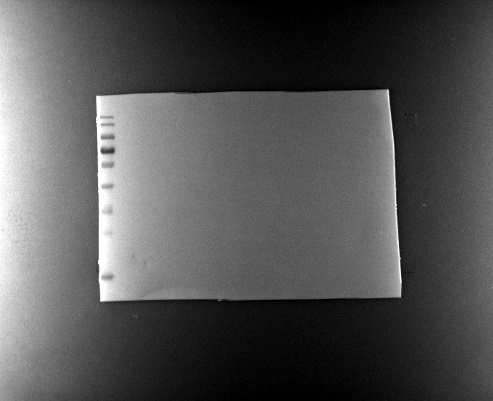


RANKL-3




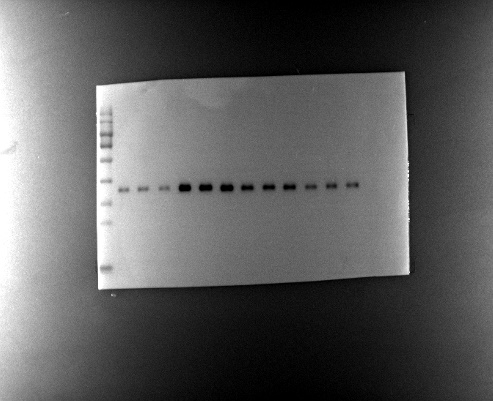

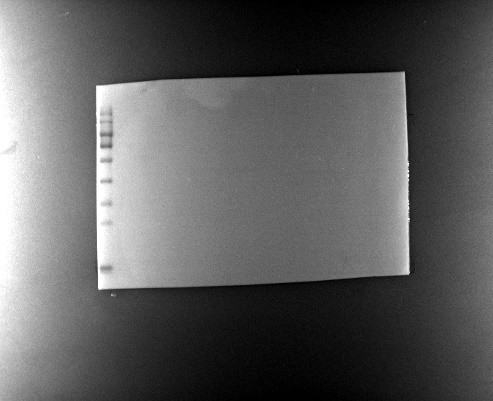


RANKL-4


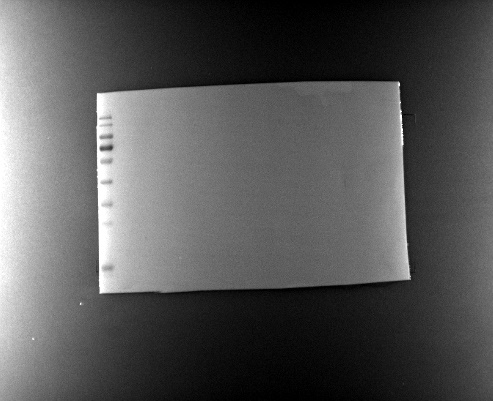

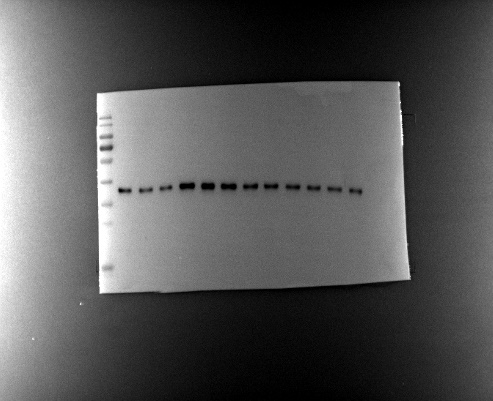




TFAM-1




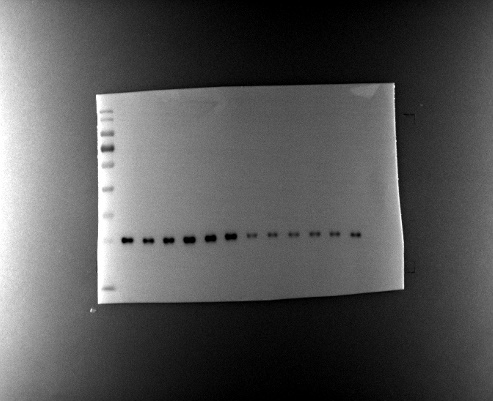

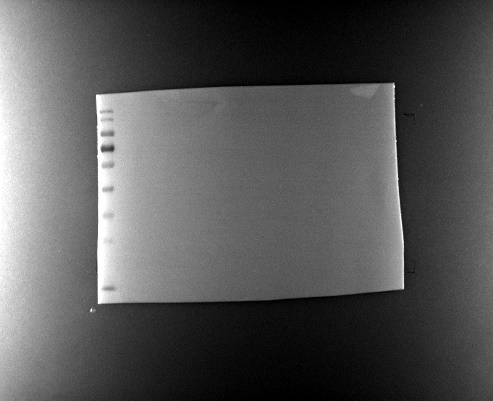


TFAM-2




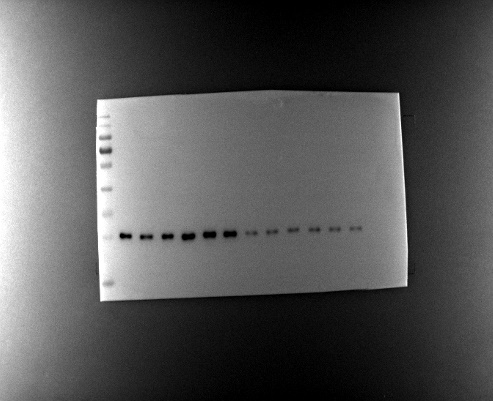

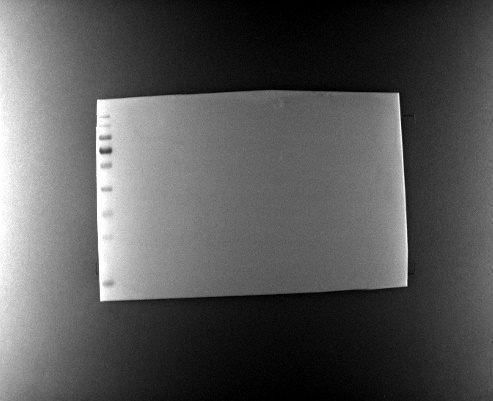


TFAM-3


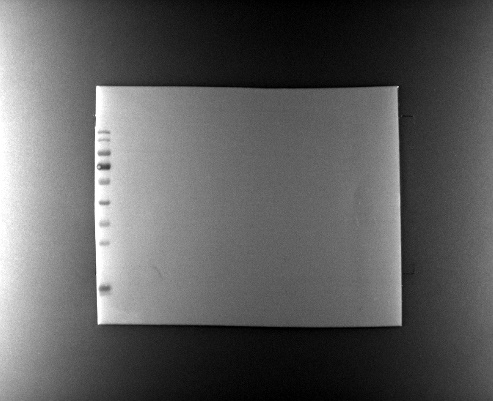

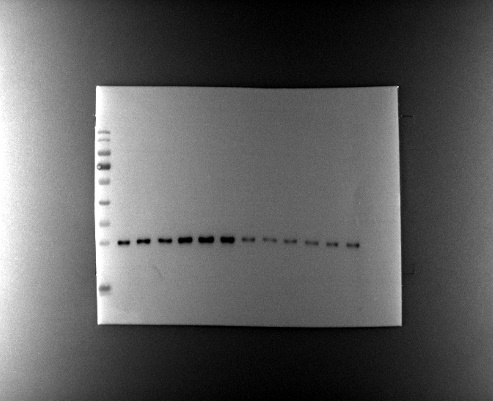




TFAM-4


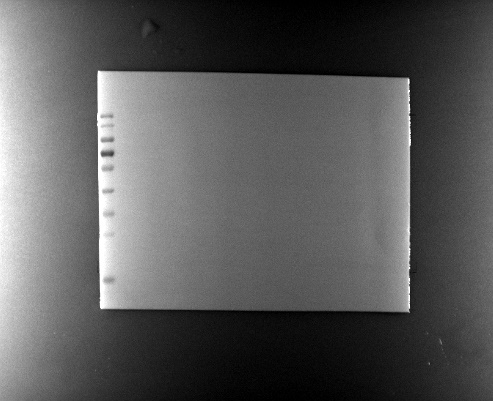

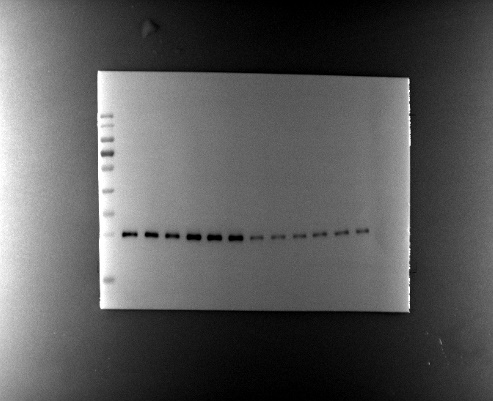




Supplementary File 3: Detailed Statistical Data for Protein and mRNA Expression Levels

| **Variable** | **Overall, N = 48^1^** | **NON-OP  N = 12 (25%)^1^** | **OP N = 36 (75%)^1^** | **statistics** | **p-value^2^** |
| --- | --- | --- | --- | --- | --- |
| **SOD2** | 180.31 [149.39, 209.78] | 243.05 [217.47, 260.73] | 167.61 [144.20, 186.96] | 413.00 | <0.001 |
| **MDA** | 5.64 (1.32) | 3.84 (0.31) | 6.25 (0.90) | 0.00 | <0.001 |
| **TFAM(PCR)** | 0.73 [0.36, 1.39] | 1.26 [0.70, 1.96] | 0.56 [0.32, 0.86] | 305.00 | 0.034 |
| **RANKL(PCR)** | 1.93 [1.24, 3.60] | 0.93 [0.66, 1.55] | 2.22 [1.69, 3.77] | 73.00 | <0.001 |
| **OPG(PCR)** | 0.14 [0.08, 0.41] | 0.98 [0.72, 1.32] | 0.10 [0.07, 0.16] | 431.00 | <0.001 |
| **RANKL/OPG(PCR)** | 15.58 [3.54, 37.75] | 1.34 [0.42, 2.35] | 25.93 [12.89, 42.63] | 0.00 | <0.001 |
| **BMP(PCR)** | 0.65 [0.36, 1.16] | 1.02 [0.48, 1.45] | 0.60 [0.31, 1.06] | 283.00 | 0.114 |
| **TFAM(WB)** | 0.47 [0.28, 0.78] | 0.83 [0.80, 0.92] | 0.30 [0.27, 0.60] | 407.00 | <0.001 |
| **RANKL(WB)** | 0.55 (0.27) | 0.25 (0.13) | 0.65 (0.22) | -5.95 | <0.001 |
| **OPG(WB)** | 0.23 [0.12, 0.56] | 0.77 [0.65, 0.83] | 0.19 [0.10, 0.28] | 430.00 | <0.001 |
| **BMP(WB)** | 0.69 [0.56, 0.74] | 0.73 [0.68, 0.76] | 0.66 [0.44, 0.72] | 307.00 | 0.030 |
| ^1^Median [IQR]; Mean (SD) | | | | | |
| ^2^Wilcoxon rank sum exact test; Wilcoxon rank sum test; Two Sample t-test | | | | | |

**Supplementary File 4: Complete Correlation Matrix Results**

**R-values corresponding to Spearman correlations**

|  | SOD2 | MDA | TFAM | RANKL | OPG | RANKL/OPG | BMP | BMD |
| --- | --- | --- | --- | --- | --- | --- | --- | --- |
| SOD2 | 1 | -0.84078 | 0.154198649 | -0.23792 | 0.412853 | -0.314511972 | 0.054958 | 0.650646 |
| MDA | -0.84078 | 1 | 0.017440407 | 0.352193 | -0.56436 | 0.373380998 | -0.11633 | -0.63122 |
| TFAM | 0.154199 | 0.01744 | 1 | 0.136551 | 0.372027 | -6.55E-05 | 0.103278 | 0.443011 |
| RANKL | -0.23792 | 0.352193 | 0.136550607 | 1 | -0.29699 | 0.669023888 | 0.129149 | -0.34587 |
| OPG | 0.412853 | -0.56436 | 0.372027125 | -0.29699 | 1 | -0.440894898 | 0.218526 | 0.655726 |
| RANKL/OPG | -0.31451 | 0.373381 | -6.55E-05 | 0.669024 | -0.44089 | 1 | -0.16489 | -0.39221 |
| BMP | 0.054958 | -0.11633 | 0.103277958 | 0.129149 | 0.218526 | -0.164887536 | 1 | 0.12945 |
| BMD | 0.650646 | -0.63122 | 0.443011353 | -0.34587 | 0.655726 | -0.392212296 | 0.12945 | 1 |

**P-values corresponding to Spearman correlations**

|  | SOD2 | MDA | TFAM | RANKL | OPG | RANKL/OPG | BMP | BMD |
| --- | --- | --- | --- | --- | --- | --- | --- | --- |
| SOD2 | 1 | 7.59E-14 | 0.295361 | 0.10345 | 0.003543 | 0.02947 | 0.710633 | 5.55E-07 |
| MDA | 7.59E-14 | 1 | 0.906341 | 0.01409 | 2.94E-05 | 0.00895 | 0.43106 | 1.51E-06 |
| TFAM | 0.295361 | 0.906341 | 1 | 0.354728 | 0.009221 | 0.999647 | 0.484839 | 0.001614 |
| RANKL | 0.10345 | 0.01409 | 0.354728 | 1 | 0.040378 | 2.01E-07 | 0.381653 | 0.016044 |
| OPG | 0.003543 | 2.94E-05 | 0.009221 | 0.040378 | 1 | 0.00171 | 0.135651 | 4.22E-07 |
| RANKL/OPG | 0.02947 | 0.00895 | 0.999647 | 2.01E-07 | 0.00171 | 1 | 0.262736 | 0.005831 |
| BMP | 0.710633 | 0.43106 | 0.484839 | 0.381653 | 0.135651 | 0.262736 | 1 | 0.380533 |
| BMD | 5.55E-07 | 1.51E-06 | 0.001614 | 0.016044 | 4.22E-07 | 0.005831 | 0.380533 | 1 |
